# Development and Validation of a Multiplex Non-HLA Antibody Assay for the Screening of Kidney Transplant Recipients

**DOI:** 10.3389/fimmu.2018.03002

**Published:** 2018-12-19

**Authors:** Elena G. Kamburova, Tineke Kardol-Hoefnagel, Bram W. Wisse, Irma Joosten, Wil A. Allebes, Arnold van der Meer, Luuk B. Hilbrands, Marije C. Baas, Eric Spierings, Cornelis E. Hack, Franka E. van Reekum, Arjan D. van Zuilen, Marianne C. Verhaar, Michiel L. Bots, Adriaan C. A. D. Drop, Loes Plaisier, Jan Meeldijk, Niels Bovenschen, Marc A. J. Seelen, Jan Stephan Sanders, Bouke G. Hepkema, Annechien J. A. Lambeck, Laura B. Bungener, Caroline Roozendaal, Marcel G. J. Tilanus, Christina E. Voorter, Lotte Wieten, Elly M. van Duijnhoven, Mariëlle A. C. J. Gelens, Maarten H. L. Christiaans, Frans J. van Ittersum, Shaikh A. Nurmohamed, Neubury M. Lardy, Wendy Swelsen, Karlijn A. M. I. van der Pant, Neelke C. van der Weerd, Ineke J. M. ten Berge, Frederike J. Bemelman, Paul J. M. van der Boog, Johan W. de Fijter, Michiel G. H. Betjes, Sebastiaan Heidt, Dave L. Roelen, Frans H. Claas, Henny G. Otten

**Affiliations:** ^1^Laboratory of Translational Immunology, University Medical Center Utrecht, Utrecht, Netherlands; ^2^Laboratory Medicine, Laboratory of Medical Immunology, Radboud Institute for Molecular Life Sciences, Radboud University Medical Center, Nijmegen, Netherlands; ^3^Department of Nephrology, Radboud Institute for Health Sciences, Radboud University Medical Center, Nijmegen, Netherlands; ^4^Department of Nephrology and Hypertension, University Medical Center Utrecht, Utrecht, Netherlands; ^5^Julius Center for Health Sciences and Primary Care, University Medical Center Utrecht, Utrecht, Netherlands; ^6^Department of Pathology, University Medical Center Utrecht, Utrecht, Netherlands; ^7^Department of Nephrology, University of Groningen, University Medical Center Groningen, Groningen, Netherlands; ^8^Department of Laboratory Medicine, University Medical Center Groningen, University of Groningen, Groningen, Netherlands; ^9^Tissue Typing Laboratory, Department of Transplantation Immunology, Maastricht University Medical Center, Maastricht, Netherlands; ^10^Division of Nephrology, Department of Internal Medicine, Maastricht University Medical Center, Maastricht, Netherlands; ^11^Amsterdam University Medical Center, Department of Nephrology, Vrije Universiteit Amsterdam, Amsterdam, Netherlands; ^12^Department of Immunogenetics, Sanquin Diagnostic Services, Amsterdam, Netherlands; ^13^Renal Transplant Unit, Department of Internal Medicine, Amsterdam University Medical Center, University of Amsterdam, Amsterdam, Netherlands; ^14^Department of Nephrology, Leiden University Medical Center, Leiden, Netherlands; ^15^Department of Internal Medicine, Nephrology, Erasmus Medical Center, Rotterdam, Netherlands; ^16^Department of Immunohematology and Blood Transfusion, Leiden University Medical Center, Leiden, Netherlands

**Keywords:** non-HLA antibody, kidney transplant, Luminex, multiplex assay, protein production, HaloTag

## Abstract

The best treatment for patients with end-stage renal disease is kidney transplantation. Although graft survival rates have improved in the last decades, patients still may lose their grafts partly due to the detrimental effects of donor-specific antibodies (DSA) against human leukocyte antigens (HLA) and to a lesser extent also by antibodies directed against non-HLA antigens expressed on the donor endothelium. Assays to detect anti-HLA antibodies are already in use for many years and have been proven useful for transplant risk stratification. Currently, there is a need for assays to additionally detect multiple non-HLA antibodies simultaneously in order to study their clinical relevance in solid organ transplantation. This study describes the development, technical details and validation of a high-throughput multiplex assay for the detection of antibodies against 14 non-HLA antigens coupled directly to MagPlex microspheres or indirectly via a HaloTag. The non-HLA antigens have been selected based on a literature search in patients with kidney disease or following transplantation. Due to the flexibility of the assay, this approach can be used to include alternative antigens and can also be used for screening of other organ transplant recipients, such as heart and lung.

## Introduction

Kidney transplantation is the preferred treatment for most patients with end-stage renal disease. Pretransplant donor-specific anti-human leukocyte antigen (HLA) antibodies (DSA) have been shown to be a major risk factor in kidney graft loss (1–3). In recent years non-HLA antibodies are increasingly being recognized as a cause for antibody-mediated graft dysfunction leading to graft loss, due to reports of antibody-mediated rejection or C4d deposition in the absence of circulating DSA (4–6). A landmark study (7) in 2005 indicated a role for non-HLA immunity by showing a relation between graft loss in HLA-identical sibling kidney transplants and percentage panel-reactive antibodies (PRA) against HLA antigens. As graft loss could not be attributed to DSA, PRA served as an indicator of increased immunity against the graft, including non-HLA antigens (7). In the past years, several reports have been published describing AMR in the absence of circulating DSA (8–17). These reports have raised interest in the identification of immunogenic non-HLA molecules and a number of target antigens were identified. At present, there is a need for the development of coherent screening assays for the detection of multiple non-HLA antibodies simultaneously in order to study their clinical relevance in solid organ transplantation (18).

Based on a literature search we have selected fourteen non-HLA targets deemed relevant for kidney transplantation (see Table 1 for a detailed description). Antibodies against angiotensin-II type I receptor (AT1R) and endothelin type A receptor (ETAR) expressed on the endothelium have already been associated with kidney graft loss using a commercially available ELISA (23, 25). The other twelve antibodies, found in patients with kidney disease or after kidney transplant, have not yet been associated with graft loss. In this study, we describe the development, technical details, and validation of a multiplex assay on a Luminex platform that can be used to detect non-HLA antibodies in kidney transplant recipients. In the PROCARE study (2) the clinical relevance of these non-HLA antibodies will be measured in 4770 sera collected prior to kidney transplantation, and the results will be reported separately. Using this high-throughput assay, the non-HLA antibody status can be determined in large cohorts and potentially be used in pre- and post-transplant risk stratification.

**Table 1:** Fourteen non-HLA proteins selected based on a literature search in patients with kidney disease or after kidney transplant.

**Protein**	**Other name**	**UniProtKB**	**Localization**	**Expression**	**References**	**Patient population**
Agrin		O00468	Secreted, extracellular matrix	Wide expression including glomerular basement membrane	(19)	Patients with transplant glomerulopathy
APMAP (Adipocyte plasma membrane-associated protein)	C20orf3	Q9HDC9	Single pass membrane protein	Ubiquitously expressed	(20)	Patients awaiting kidney retransplant
ARHGDIB (Rho GDP-dissociation inhibitor 2)	RhoGDI2	P52566	Intracellular: cytoplasm	Wide expression including renal pelvis and glomerulus	(21)	Chronic hemodialysis patients
ARHGEF6 (Rho guanine nucleotide exchange factor 6)		Q15052	Intracellular: cytosol	Ubiquitously expressed	(22)	Pediatric kidney transplant recipients
AT1R (Angiotensin type I receptor)		P30556	Transmembrane protein	Adipose and soft tissues	(5, 23)	Kidney transplant recipients
Endorepellin (C-terminal fragment of perlecan)		P98160	Secreted, extracellular matrix	Ubiquitously expressed	(24)	Kidney transplant recipients with acute vascular rejection
ETAR (Endothelin type A receptor)	EDNRA	P25101	Transmembrane protein	Ubiquitously expressed	(25))	Kidney transplant recipients
Lamin B1		P20700	Intracellular: Nuclear membrane	Ubiquitously expressed	(21)	Chronic hemodialysis patients
LPLUNC1 (BPI fold-containing family B member 1)	BPIFB1/C20orf114	Q8TDL5	Secreted, extracellular matrix	Respiratory epithelia, stomach, small intestine and salivary gland	(20)	Patients awaiting kidney retransplant
PECR (Peroxisomal trans-2-enoyl-CoA reductase)		Q9BY49	Intracellular: cytoplasm, peroxisome	Ubiquitously expressed	(26)	Patients with transplant glomerulopathy
PLA2R (Phospholipase A2 receptor)		Q13018	Membrane, secreted	Renal glomeruli	(27)	Patients with membranous nephropathy
PRKCZ (Protein kinase C zeta type)		Q05513	Intracellular: Cytoplasm	Ubiquitously expressed	(28)	Pediatric kidney transplant recipients
Tubb4B (Tubulin beta-4B)		P68371	Intracellular: cytoplasm	Ubiquitously expressed	(21)	Chronic hemodialysis patients
Vimentin		P08670	Intracellular: cytoplasm	Ubiquitously expressed	(21, 29)	Chronic hemodialysis patients and renal transplant recipients with IFTA (interstitial fibrosis and tubular atrophy)

## Materials and Methods

### In-house Protein Production

For the production of the HaloTag-proteins, we generated a universal vector containing the HaloTag® open reading frame [from the HaloTag® CMV*d1* Flexi® Vector (Promega, Madison, Wisconsin)] cloned in a pcDNA3.1 derived plasmid (Thermo Fisher Scientific, Waltham, Massachusetts) with multiple cloning site altered and added woodchuck posttranscriptional regulatory element (WPRE) before the poly A tail in order to stabilize the mRNA and enhance protein production (30). For PLA2R without a HaloTag we used the pcDNA3.1-WPRE vector only. We ordered the different inserts each starting with a BglII restriction site, then a Kozak sequence and a standard signal peptide, the codon optimized DNA sequence coding for the protein of interest, followed by 6-Histidines for purification purposes and ending with an XhoI restriction site from the Invitrogen GeneArt Gene synthesis (Thermo Fisher Scientific) (Figure 3). Using the BglII and XhoI restriction sites we transferred the synthesized inserts from the suppliers vector to our HaloTag expression vector (Figure 3). The amino acid sequence used for the in-house produced proteins are provided in Supplementary Table S1. For all constructs the complete open reading frames were sequence verified by Sanger sequencing. Subsequently, 1 x 10^8^ HEK293-F cells were co-transfected with a 100 μg DNA mix (containing per non-HLA an optimized ratio of the pAdVAntage^TM^ vector (Promega) and the vector containing the sequence of the protein of interest) and 130 μg 293-Fectin in FreeStyle™ 293 Expression Medium (both Thermo Fisher Scientific). As the universal vector contained a signal peptide, the proteins were secreted into the culture supernatant, which was harvested at day 4. An AKTA start protein purification system (GE Lifesciences, Chicago, Illinois) fitted with a HisTrap^TM^ HP column (GE Lifesciences) was used to purify proteins from the cell supernatants. The fractions containing the protein of interest were pooled. To remove imidazole and other contaminants smaller than 10 kDa, the pooled fractions were dialyzed to PBS in a Slide-A-Lyzer™ Dialysis Cassette 10 kDa (Thermo Fisher Scientific). The proteins were detected by Western blot using various antibodies. See Supplementary Table S2 for a list of all antibodies used in the Western blot and their dilutions.

### Treatment With PNGase F

All purchased and in-house produced proteins with an N-Acetylglucosamine (N-GlcNAc) according to UniProt (Table 2) were treated with PNGase F (New England Biolabs) according to manufacturer's protocol. The PNGase F treated and untreated proteins were detected by Western blot using various antibodies (Supplementary Table S2).

**Table 2:** Overview of all purchased and in-house produced proteins.

**Coupling**	**Protein**	**Source**	**Company**	**Size including Tags (kDa)**	**N-GlcNAc**	**Glycosylation confirmed by PNGase F treatment**
Direct	IgG	Human serum	Sigma Aldrich	150	**–**	**–**
	Agrin	CHO cell line	R&D systems	100	No	–
	APMAP	Wheat Germ (*in vitro*)	Abnova	73	Yes	No
	ARHGDIB	Wheat Germ (*in vitro*)	Abnova	48	No	–
	ARHGEF6	Wheat Germ (*in vitro*)	Abnova	114	No	–
	AT1R	Wheat Germ (*in vitro*) with proprietary liposome technology	Abnova	41	Yes	No
	Endorepellin	Mouse myeloma cell line	R&D systems	90	Yes	Yes
	ETAR	Wheat Germ (*in vitro*)	Abnova	75	Yes	No
	LMNB1	Wheat Germ (*in vitro*)	Abnova	93	No	–
	LPLUNC1	HEK293 Cells	Sino Biological Inc.	53	Yes	Yes
	PECR	E coli	Abcam	35	No	–
	PLA2R	HEK293 Cells	in-house production	160	Yes	Yes
	PRKCZ	Wheat Germ (*in vitro*)	Abnova	94	No	–
	Transferrin	Serum of non-immunized animals	Jackson ImmunoResearch	80	No	–
	TUBB4B	Wheat Germ (*in vitro*)	Abnova	75	No	–
	Vimentin	E coli	R&D systems	55	No	–
HaloTag	Agrin_HaloTag	HEK293 cells	in-house production with HaloTag	138	No	–
	APMAP_HaloTag	HEK293 cells	in-house production with HaloTag	85	Yes	Yes
	ARHGDIB_HaloTag	HEK293 cells	in-house production with HaloTag	59	No	–
	ARHGEF6_HaloTag	HEK293 cells	in-house production with HaloTag	126	No	–
	*AT1R_HaloTag*	*NA*	*NA*	–	–	–
	Endorepellin_HaloTag	HEK293 cells	in-house production with HaloTag	113	Yes	Yes
	ETAR_HaloTag	HEK293 cells	in-house production with HaloTag	52	Yes	Yes
	LMNB1_HaloTag	HEK293 cells	in-house production with HaloTag	105	No	–
	LPLUNC1_HaloTag	HEK293 cells	in-house production with HaloTag	91	Yes	Yes
	*PECR_HaloTag*	*NA*	*NA*	–	–	–
	PLA2R_HaloTag	HEK293 cells	in-house production with HaloTag	197	Yes	Yes
	PRKCZ_HaloTag	HEK293 cells	in-house production with HaloTag	106	No	–
	Transferrin_HaloTag	HEK293 cells	in-house production with HaloTag	114	No	–
	TUBB4B_HaloTag	HEK293 cells	in-house production with HaloTag	89	No	–
	Vimentin_HaloTag	HEK293 cells	in-house production with HaloTag	96	No	–

*NA, not available, N-GlcNAc, N-Acetylglucosamine; see Table 1 for the full protein names and additional information*.

### Direct Coupling of Proteins to Microspheres

**Figure 1:**
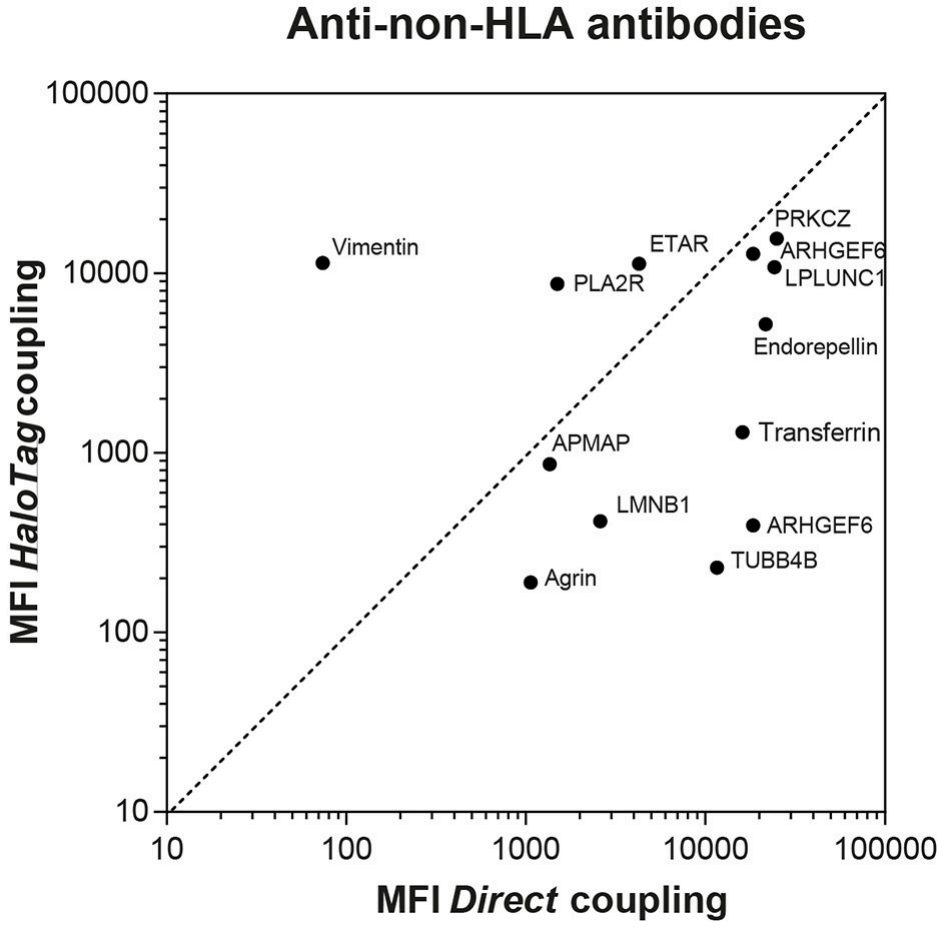
Comparison between directly and HaloTag coupled microspheres after incubation with commercially available animal anti-non-HLA antibodies. For AT1R and PECR only microspheres with direct coupling were available, therefore the comparison with HaloTag coupled microspheres cannot be depicted. The MFI measured after incubation of the AT1R microspheres with the anti-AT1R antibody was 13,760 and 18,842 for the PECR microspheres after incubation with the anti-PECR antibody.

First, using a Vivaspin® 500 (Sartorius, Göttingen, Germany) with pore size 10 kDa, 10 of the 15 purchased proteins which were dissolved in glycerol or Tris-HCl were transferred to PBS. Covalent coupling of the sixteen (proteins Table 2 upper part) different carboxylated MagPlex® Microspheres (Luminex Corp, Austin, Texas) each with different emitting fluorescence pattern was performed by following the procedures recommended by Luminex (31). In short, 6.25 × 10^6^ microspheres (500 μl of stock) suspension was resuspended in microtiter tubes containing 0.1 M sodium phosphate butter (pH 6.1) to a final volume of 200 μl. Fresh solutions of N-hydroxy-sulfosuccinimide (Sulfo-NHS) and 1-ethyl-3-(3-dimethylaminopropyl)-carbodiimide hydrochloride (Pierce), both at 50 mg/ml, were prepared in phosphate buffer, and 25 μl of each solution was sequentially added to stabilize the reaction and activate the microspheres. This suspension was incubated for 5 min at room temperature and then resuspended in 625 μl protein solution (1.5 μg protein per 1 × 10^6^ microspheres). The mixture was incubated overnight in the dark with continuous shaking. The microspheres were then incubated with 625 μl PBS-0.05% Tween-20 for 4 h. After aspiration, the microspheres were blocked with PBS-0.1% BSA-0.1% sodium azide to a final concentration of 2,500 beads/μl. The microspheres were counted on Beckman Coulter Counter and stored in the dark at 4°C. Coupling efficiency was tested using anti-protein antibodies (Figure 1 and Supplementary Table S2).

### Coupling of HaloTag Proteins to Microspheres

First, microspheres were coupled with HaloTag Amine (O4) Ligand (Promega). 6.25 × 10^6^ microspheres were suspended in a solution of 100 mmol/l MES (pH 6.0) containing 5 mg/ml 1-ethyl-3-(3-dimethylaminopropyl)-carbodiimide hydrochloride (Pierce) and 0.2 mg HaloTag Amine Ligand. After 2 h incubation in the dark, the microspheres were washed and resuspended in 100 mmol/l MES (pH 4.5) and stored at 4°C for at least 16 h before using for protein coupling. Next, after aspiration the microspheres coupled with HaloTag amine ligand were washed with PBS-0.05% Tween-20. This suspension was incubated with 1 ml HaloTag-protein fraction (protein concentration ranging from 25 to 100 μg/ml) for 1 h in the dark with continuous shaking. After washing with PBS-0.05% Tween-20, the microspheres were aspirated and blocked with PBS-0.1% BSA-0.1% sodium azide to a final concentration of 2,500 beads/μl. The microspheres were counted on Beckman Coulter Counter and stored in the dark at 4°C. Coupling efficiency was tested using anti-protein antibodies (Figure 1 and Supplementary Table S2).

### Multiplex Luminex Assay

A mix of 31 different microspheres was made consisting of the 15 directly-coupled proteins, 13 in-house produced HaloTag-coupled proteins, the IgG-coupled microsphere as a positive control, the HaloTag amine-coupled microsphere, and an empty microsphere. 1,500 microspheres per well were used of each specificity diluted in PBS−0.1% BSA (wash buffer). First, 48 μl diluted microsphere mix was aliquoted into a 96-well Bio-Plex Pro Flat bottom plate (Bio-Rad, Hercules, California) and then 2 μl serum was added (serum dilution 1:25). For assay validation, instead of serum anti-protein antibodies were added. After overnight incubation in the dark at room temperature with continuous shaking, samples were washed using a Bio-Plex Pro Wash station (Bio-Rad). Next, 50 μl of 1:50 R-phycoerythrin-conjugated goat-anti human antibody (Jackson ImmunoResearch, Cambridgeshire, UK) diluted in wash buffer was added per well. After 30 min incubation at room temperature with continuous shaking, 50 μl wash buffer was added and samples were measured on a Luminex 200 flow analyzer (Luminex Corp). See Supplementary Table S3 for a list of all antibodies used in the Luminex assay and the used dilution. Readout is provided as median fluorescence intensity (MFI).

### Commercial Autoantibody Luminex Assay

For comparison, LABScreen Autoantibody assay group 1 (Lot 1: 32 targets) and group 2 (Lot 1: 1 target) from One Lambda (Canoga Park, CA) was used to determine the non-HLA antibody profile in patient sera according to manufacturer's instructions. One Lambda donated reagents but was not involved in either the conduct of the study or the preparation of the manuscript. In brief, 10 μl of Autoantibody microsphere mix was incubated with 40 μl of serum in a 96 well V-bottom plate for 30 min in the dark at room temperature with continuous shaking. After washing, PE-conjugated anti-human IgG was added and samples were again incubated for 30 min in the dark at room temperature with continuous shaking. Finally, samples were washed again, PBS was added and samples were measured on a Luminex 200 flow analyzer. Readout is provided as median fluorescence intensity (MFI) and adjusted for background by subtracting the MFI of the negative control microsphere from the MFI of the specific microsphere. This adjusted value is referred to as Baseline.

### Serum Samples

The use of sera and experimental protocols was approved by the Research Ethics Committee for Biobanks and the Medical Ethics Committee of the University Medical Center Utrecht and was performed in accordance with the FEDERA Code of Conduct. Sera were passed through a 96-well 1.2 μm MultiScreen filter plate (Millipore, Billerica, MA) to clear debris before used in the Luminex assay. No other serum pretreatment was performed.

Eighteen patient sera from a national inter-laboratory comparison of the qualitative anti-PLA2R indirect immunofluorescence test (IIFT) were used for validation of our PLA2R Luminex assay. The national consensus conclusions were used to compare to MFI levels we measured. Sera of 87 controls (healthy persons fit to work or deceased donors approved for lung donation) were used to assess non-HLA antibodies in persons without end-stage renal disease. From a national consortium study (2), we selected eight sera from patients with end-stage renal disease with high MFI levels against a variety of non-HLA specificities to test their specificity in inhibition experiments using each produced non-HLA antigen added in fluid phase. From the same consortium study, fourteen sera were selected containing antibodies against 8 non-HLA proteins both present in our assay and the commercially available assay in order to compare results of our assay to the commercially available Autoantibody assay.

### Statistical Analysis

Statistical analyses were performed with SAS 9.4 (SAS Institute, Cary, NC, United States). Continuous data were analyzed with the Mann-Whitney *U*-test. The correlation between our assay and the commercially available Autoantibody assay was evaluated using Pearson's correlation coefficient. P < 0.05 was considered as statistically significant.

## Results

### Protein Production

**Figure 2:**
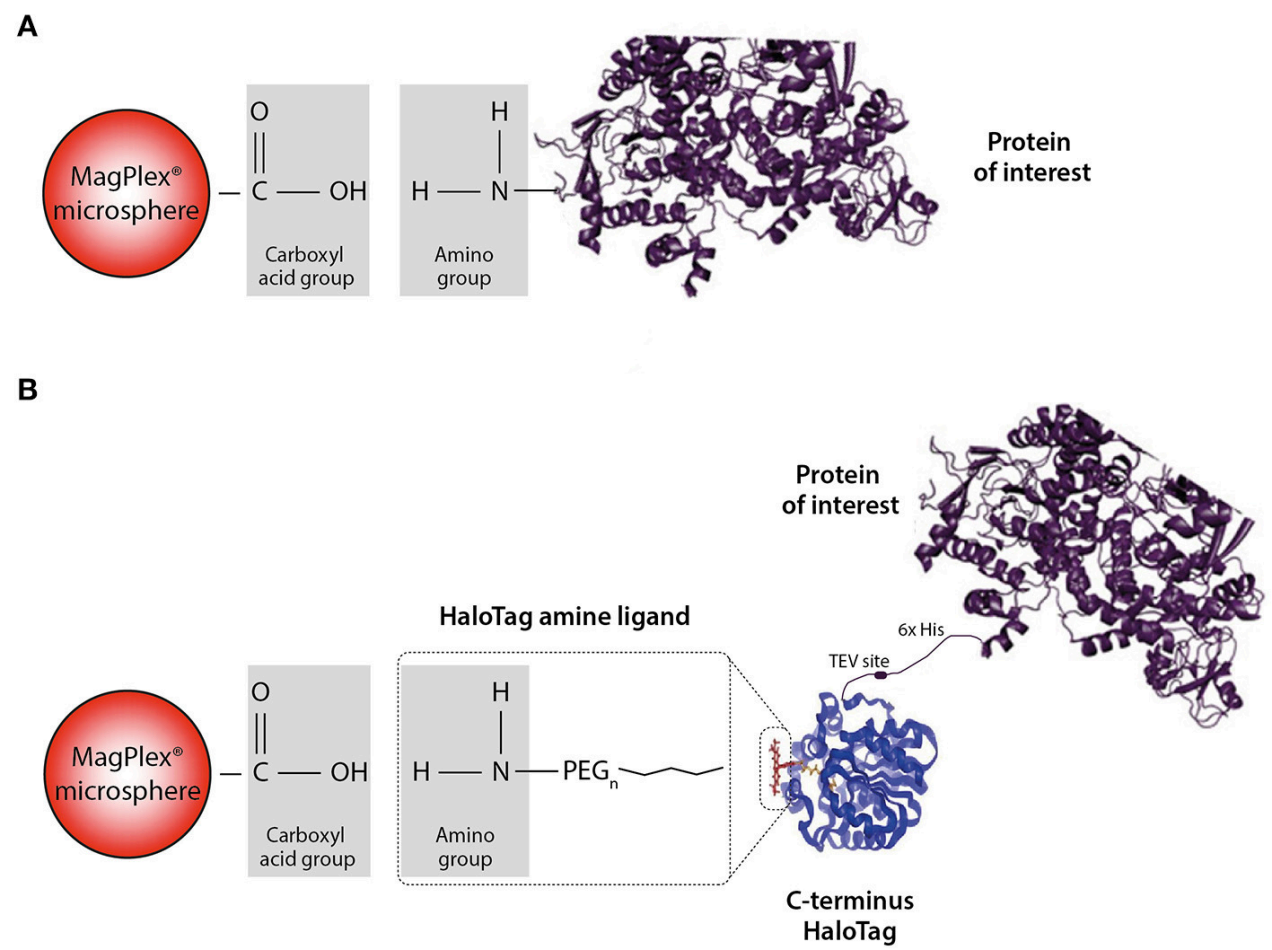
Schematic illustration of the direct and HaloTag coupling to the MagPlex microspheres**. (A)** We refer to direct coupling when the amino group of the protein is directly coupled to the carboxylated microsphere. **(B)** For the HaloTag coupling a HaloTag amine ligand serves as a connection between the carboxyl group and the HaloTag of the protein. Due to this indirect coupling, the protein of interest is freely accessible compared to the direct situation. Part of the figure is adapted from Promega.

We chose two different strategies to covalently couple the protein antigens to Luminex microspheres: (1) direct coupling via the amino group of the protein to the carboxyl group on the microsphere (Figure 2A) and (2) indirect (HaloTag) coupling via the HaloTag-recombinant protein to the HaloTag amine ligand bound to the microsphere (Figure 2B). Both coupling strategies result in covalent and stable binding of the proteins to the microspheres. For direct coupling we purchased all non-HLA proteins, except PLA2R (produced in-house), and for the coupling via the HaloTag we produced all non-HLA proteins in-house. Table 2 provides an overview of all proteins with their production method or source, size including Tags, whether N-Acetylglucosamine (N-GlcNAc) was present according to UniProt, and whether glycosylation could be confirmed by treatment with PNGase F. We were not able to produce AT1R and PECR in-house because the proteins were cleaved before excretion into the culture supernatant. Human IgG coated on microspheres was used as a positive control, and there were four negative control microspheres: (1) empty, (2) coated with only HaloTag amine ligand, (3) direct-coupled transferrin, and (4) HaloTag-coupled transferrin as autoantibodies against transferrin have not been reported in end-stage renal disease or after kidney transplantation.

**Figure 3:**
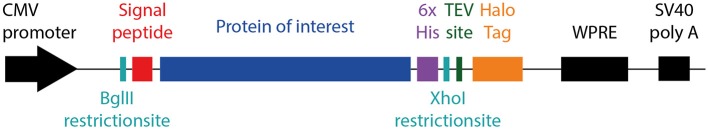
Schematic illustration of a part of the universal vector used for the production of HaloTag proteins. In the general pcDNA3-WPRE vector starting with a CMV promoter and including the HaloTag sequence and the WPRE sequence, which enhances expression, we ligated an insert consisting of a BglII restriction site, then a signal peptide, the DNA sequence of the protein of interest, followed by 6-Histidines and ending with an XhoI restriction site.

**Figure 4:**
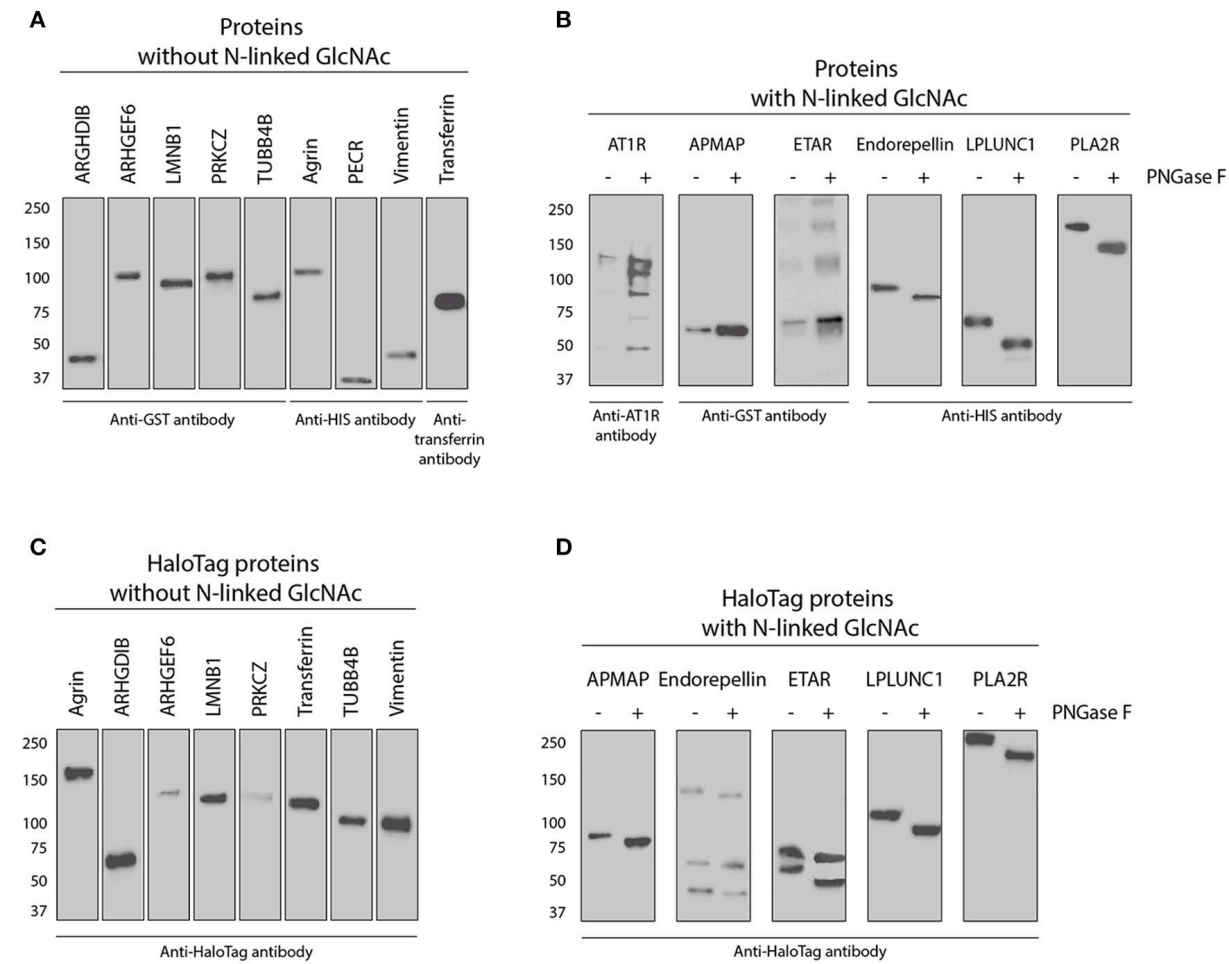
Western blot of all purchased and in-house produced proteins used for direct- and HaloTag coupling to microspheres. **(A)** Nine purchased proteins without an N-Acetylglucosamine (N-GlcNAc) according to UniProt (see also Table 2). Proteins were detected with either tag or protein specific antibodies as indicated. **(B)** Five purchased and one in-house produced proteins (PLA2R) have an N-GlcNAc according to UniProt and were therefore treated with PNGase F to check for this glycosylation. Proteins before and after treatment were detected with either tag or protein specific antibodies as indicated. **(C)** Eight in-house produced HaloTag proteins without a GlcNAc according to UniProt were all stained with anti-HaloTag antibody. **(D)** Five-in-house produced HaloTag proteins with an N-GlcNAc according to UniProt were treated with PNGase F to check for this glycosylation. These proteins were all detected before and after treatment with anti-HaloTag antibody.

All in-house produced proteins were generated using a universal cloning vector including a C-terminal HaloTag and an insert containing the sequence of the protein of interest (see Figure 3 for the vector design). The AKTA start protein purification system was used to remove potential (small) contaminants such as the HaloTag protein (34 kDa) which does not contain histidine residues. As we produced relatively low amounts of proteins, we were not able to check for purity by Coomassie blue protein staining. The HaloTag coupling to the microspheres is another purification step, as HaloTag is usually used for protein purification (as advertised by Promega). Therefore, protein size and glycosylation status were studied by Western blot using appropriate antibodies (Figure 4) and compared to the predicted size (Table 2). The estimated size was comparable to the predicted size for all 28 proteins. Glycosylation could not be demonstrated for three of six purchased and directly-coupled proteins with expected N-GlcNAc (APMAP, AT1R, and ETAR), since the size of proteins was unchanged after treatment with PNGase F (Figure 4B). The Western blot of the purchased AT1R, purchased ETAR (both Figure 4B) and in-house produced ETAR_HaloTag (Figure 4D) showed multiple bands which might be caused by the presence of SDS-PAGE and DTT stable aggregates. For the three other proteins with N-GlcNAc used for direct coupling (Endorepellin, LPLUNC1 and PLA2R) glycosylation could be confirmed by PNGase F treatment. These proteins were produced in a mouse myeloma cell line or in HEK293 cells. Also for all five HaloTag proteins with N-GlcNAc we could confirm the glycosylation (Figure 4D).

### Validation of the Multiplex Non-HLA Antibody Assay

After analysis of all proteins using Western blot as described above, the proteins were coupled to MagPlex microspheres. In order to optimize detection of non-HLA antibodies, we tested different incubation times, incubation temperatures and serum dilutions. Overnight incubation at room temperature using a serum dilution of 1:25 resulted in the highest specific MFI levels compared to minimal background signal using anti-PLA2R IIFT positive sera (data not shown). Similar results were observed for patient sera containing anti-Vimentin antibodies.

**Figure 5:**
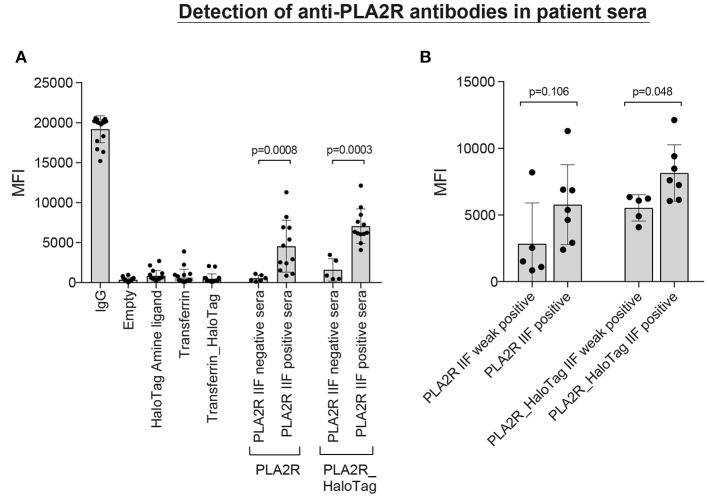
Detection of anti-PLA2R antibodies in patient sera**. (A)** 18 patient sera [6 negative and 12 positive according to the qualitative anti-PLA2R indirect immunofluorescence test (IIFT)] were incubated with a microsphere mix consisting of empty microspheres and microspheres coupled with HaloTag amine ligand, Transferrin (direct-coupling), Transferrin_HaloTag (together four negative controls), coupled with IgG (positive control), and also two specific microspheres coupled with PLA2R (direct) or PLA2R_HaloTag. The MFI values of each independent patient serum are depicted including the mean and standard deviation. **(B)** The 12 IIFT positive patient sera can be divided into weak positive (*n* = 5) and positive (*n* = 7) according to the immunofluorescence staining. Using this stratification, we plotted the MFI values measured in our assay with the two different PLA2R microspheres (direct- and HaloTag-coupling).

To assess whether the PLA2R microspheres were correctly coupled, we used patient sera with anti-PLA2R antibodies determined with the qualitative IIF test (Figure 5). The average MFI ± SD for the PLA2R directly-coupled microspheres was 548 ± 390 for the PLA2R IIFT negative sera and 4,548 ± 3,256 for the PLA2R IIFT positive sera (*P* = 0.0008). For the PLA2R_HaloTag microspheres this was 1,593 ± 1,408 for the PLA2R IIFT negative sera and 7,058 ± 2,152 for the PLA2R IIFT positive sera (*P* = 0.0003; Figure 5A). Even thought, there is a high variation between the patient sera, the PLA2R IIFT positive and negative sera could easily be distinguished based on the measured MFI of our assay. According to the qualitative IIF test, PLA2R positive sera could be subdivided into weak positive and positive. Using this stratification, we plotted the MFI values measured in our assay with the two different PLA2R microspheres (direct and HaloTag coupling). The MFI values of the positive sera were higher compared to the weak positive sera (*P* = 0.106 for PLA2R weak positive compared to PLA2R positive, and *P* = 0.048 for PLA2R_HaloTag weak positive compared to PLA2R_HaloTag positive). In our assay we defined that the MFI of the IgG microspheres should be at least 10,000 to assure correct secondary antibody binding, in line with the commercially available Luminex assay to determine HLA antibodies. The four negative control microspheres had overall low MFI values (empty microsphere average MFI ± SD: 360 ± 247; Transferrin: 692 ± 961; HaloTag amine ligand 844 ± 673; Transferrin_HaloTag 479 ± 604). 10 PLA2R IIF positive sera were used to determine assay variation on several days in our laboratory using the same lot. The mean coefficient of variation was 15.1% for the MFI of PLA2R and PLA2R_HaloTag microspheres (data not shown).

Human sera with antibodies against the other non-HLA targets are not commercially available nor present in external proficiency testing, as is the case for anti-PLA2R antibodies. To test whether coupling of the microspheres was successful, we used commercially available anti-non-HLA antibodies (Supplementary Table S3). As the principle of ELISA is comparable to our Luminex assay, we selected animal source anti-non-HLA antibodies that were validated for ELISA. We obtained validated antibodies from animal sources for 9 of the 15 non-HLA targets (Supplementary Table S3). So 6 of these specific antibodies from animal sources were not validated for the use in an ELISA assay and could therefore results in lower MFI values. Using these antibodies we compared the MFI values for the directly- and HaloTag-coupled microspheres (Figure 1). For most of the microspheres we observed relatively high MFI values, confirming the coupling and recognition of the non-HLA proteins. Potential causes of relatively low MFI values for some microspheres could be due to the fact that the antibody is not suitable for ELISA/Luminex, that the epitopes these antibodies recognize are not accessible on the direct- or HaloTag-coupled microspheres, or that the coupling efficiency is low. To check whether the signals were specific, we preincubated 8 patient sera, with high MFI levels against a variety of non-HLA specificities, with the specific protein or with transferrin (concentrations ranging from 0 to 60 ng/μl). Inhibition in antibody binding (decrease in MFI) was observed if sera were pre-incubated with the specific protein, whereas no inhibition was found after preincubation with transferrin (data not shown).

### Multiplex Assay With Healthy Control Sera

**Figure 6:**
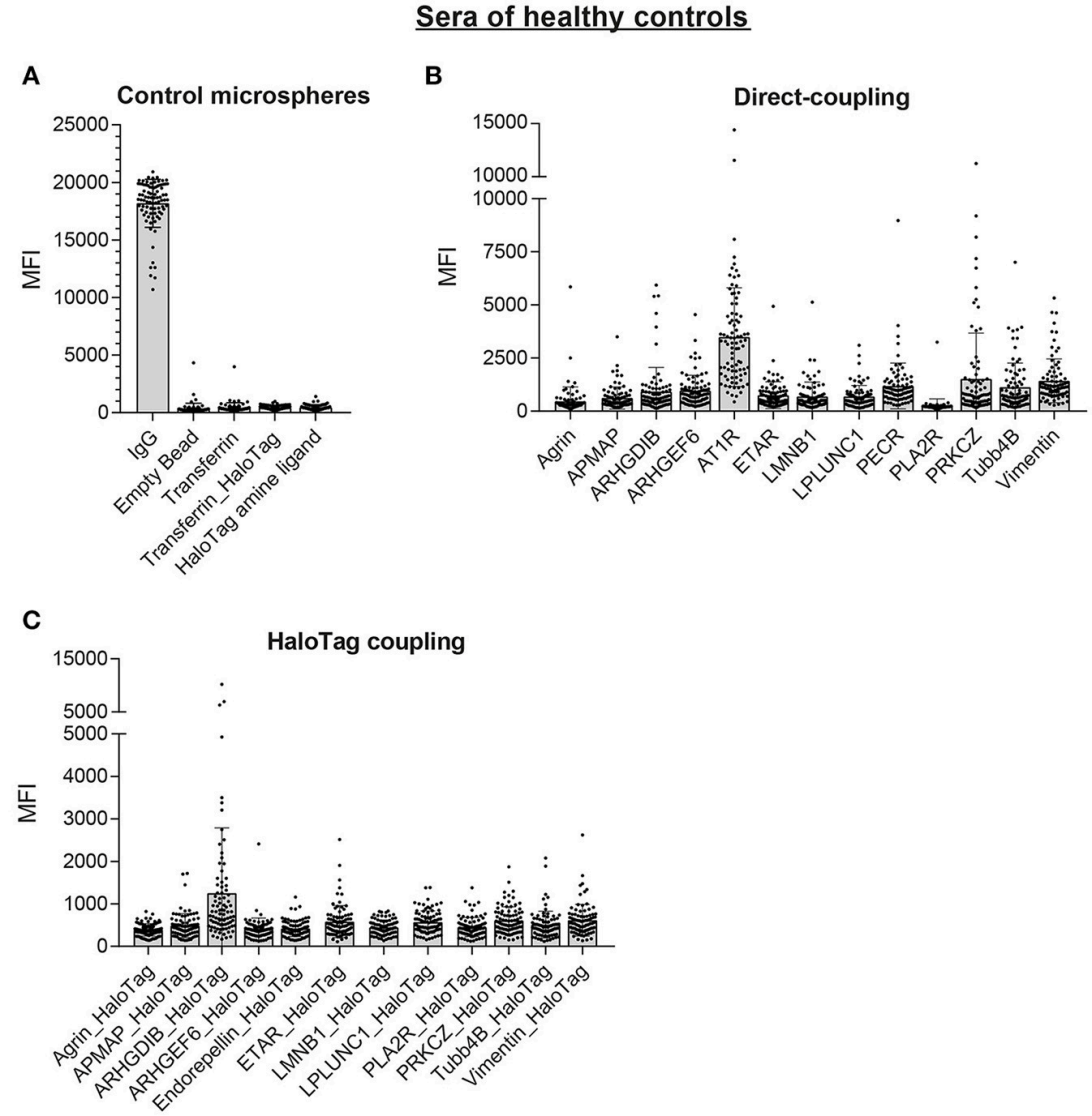
Multiplex non-HLA antibody assay using sera of 87 healthy controls. Shown are the individual MFI values with the mean ± standard deviation (SD) for the positive control microsphere (IgG) and the 4 negative control microspheres **(A)**, the 13 direct-coupled microspheres **(B)**, and the 12 HaloTag coupled microspheres **(C)**.

Next, we screened sera of 87 healthy controls with the multiplex non-HLA antibody assay. The control microspheres performed as expected: IgG-MFI was above 10,000 and the 4 negative control microspheres showed in general low MFI values (mean MFI < 500; Figure 6A). For the direct-coupled microspheres, 8 targets had a mean MFI below 1,000 (Agrin, APMAP, ARHGDIB, ARHGEF6, ETAR, LMNB1, LPLUNC1, and PLA2R), 4 targets had a mean MFI between 1,000 and 1,500 (PECR, PRKCZ, TUBB4B, and Vimentin; Figure 6B). AT1R had a mean MFI ± SD of 3,489 ± 2,322. The results acquired with the direct-coupled Endorepellin are not reliable and were excluded from further analyses. For the direct-coupled Endorepellin, the majority of microspheres had a relatively high MFI with a mean MFI ± SD of 11,769 ± 7,492. This Endorepellin was produced in a mouse cell line, and the high MFI-values appeared due to cross-reactivity of our secondary goat anti-human antibody to mouse (data not shown). For the HaloTag coupled microspheres, the mean MFI values for the healthy controls were much lower compared to the direct-coupled microspheres, except for ARHGDIB and PLA2R (Figure 6C). Most mean MFI values for the HaloTag coupled microspheres were below 1,000, only the mean MFI of ARHGDIB_HaloTag was above 1,000 (mean MFI ± SD: 1,255 ± 1,532).

### Comparison With Commercially Available Autoantibody Assay

Recently, a commercial Autoantibody multiplex assay became available which included 8 of the 14 non-HLA targets we selected (Agrin, ARHGDIB, Endorepellin (C-terminal part LG3), LMNB1, PECR, PLA2R, PRKCZ, and Vimentin). 14 patient sera were selected based on a variety of MFI levels against the 8 targets in our assay. We analyzed these sera with our direct- and HaloTag-coupled non-HLA antibody assay and compared the baseline calculations to the commercially available autoantibody assay. The correlation (r^2^) of the baseline calculations between our assay and the commercial assay varied between 0.0903 and 0.9628 (Table 3). Although it is reassuring to see that for some non-HLA targets there is a high correlation between our assay and the commercial assay, it is difficult to properly compare the assays because essential details from the commercial assay are unknown such as protein size, glycosylation, source, purity and coupling procedure to the microspheres.

**Table 3:** Correlation between the baseline calculations of our direct- and HaloTag-coupled multiplex assay to the commercially available Autoantibody assay.

**Coupling**	**Protein**	***N***	***r*^**2**^ compared to commercial assay**
Direct	Agrin	14	0.9628
	ARHGDIB	14	0.4777
	LMNB1	14	0.2635
	PLA2R	14	0.5557
	PRKCZ	14	0.5023
	Vimentin	14	0.1133
HaloTag	Agrin	14	0.0903
	ARGHDIB	14	0.4984
	Endorepellin (LG3)	14	0.2741
	LMNB1	14	0.0243
	PECR	14	0.4680
	PLA2R	14	0.9371
	PRKCZ	14	0.2633
	Vimentin	14	0.0940

*Baseline is MFI of the specific microsphere minus the MFI of negative control microsphere. r^2^, Pearson Correlation*.

## Discussion

The major objective of this study was to develop a high-throughput non-HLA antibody assay that can be used for the screening of kidney, heart and lung transplant recipients. At present, multiple commercial assays are available containing tests to measure non-HLA antibodies against a single target. These tests often lack details preventing in-house replication of the manufacturing process. In this study, we transparently provide all important details needed for the production of reagents and development of the assay for non-HLA antibody measurement and also mention individual failure in antigen production. Using the information provided here, we believe that other researchers should be able to reproduce this assay. The selection of the fourteen non-HLA targets was hypothesis-driven based on a literature search on the relation of non-HLA antibodies and kidney function. The MFI values of the PLA2R IIF positive sera were higher compared to the weak positive sera, suggesting that our Luminex assay could be used as a semi-quantitative diagnostics assay, which might also be helpful for monitoring of treatment or early detection of relapse in patients with membranous nephropathy. Although we could only independently validate the assay with patient sera containing anti-PLA2R determined with the qualitative IIF test, we expect that the production and development process is optimal for the other non-HLA targets as well, as we could confirm this using Western blot and inhibition assays. Using this multiplex assay the non-HLA antibody status of a patient can easily be determined within 24 h with only 2 μl of serum needed.

In this study we coupled non-HLA antigens directly to the microspheres as well as indirectly via a HaloTag linker. At present it is unknown which epitopes are predominantly recognized on each of the non-HLA antigens included, but as the coupling process may influence the epitopes recognized, our rationale to include both coupling procedures was to empirically find out which coupling procedure is best suited to detect clinically relevant non-HLA antibodies. To have all relevant epitopes exposed upon coupling of proteins to the microspheres, we anticipate that the HaloTag is superior to the direct-coupling, although the trade-off is that less molecules can be bound to individual microspheres resulting in less antibody binding leading to lower MFI levels.

In addition, whilst time-consuming, the HaloTag system is more flexible compared to direct coupling, allowing research groups to produce and develop their own assay with any protein of interest. Another major disadvantage of the direct-coupled assay is the dependency on commercially available proteins, (either full length proteins or peptides), and the cell lines in which they are produced can have an effect on posttranslational modifications such as glycosylation. For three purchased proteins we could not demonstrate glycosylation. This lack of glycosylation is probably explained by the *in vitro* production in wheat germ (Abnova). As we don't know the production details of the proteins used in the commercially available Autoantibody assay, the comparison between the assays is probably not completely valid.

To determine whether a serum sample is positive or negative for a non-HLA antibody, a clinically meaningful cut-off has to be defined. The commercial autoantibody multiplex assay used for comparison in this study makes use of serum samples from non-transplanted individuals. Using the results of 100 individuals, the reference background value was calculated from the median of the MFI plus 2 times the SD defining ~3–5% of healthy as being positive for non-HLA antibodies. However, this is by definition not a clinically relevant cut-off as every healthy individual potentially has antibodies against autoimmune targets, and the definition used is unrelated to clinical key-parameters such as graft loss or antibody-mediated rejection. Patients with end-stage renal disease have massive cellular damage in their kidneys and occurrence of cell death or apoptosis is known to result in release of intracellular proteins which are normally not accessible by antibodies. This release likely results in a boost of autoantibody formation generated to clear these antigens from the circulation, thus including a healthy control cohort as reference for non-HLA antibody levels for patients with end-stage renal disease may not be optimal. In our opinion, the best way to determine the clinically relevant cut-off for kidney transplant recipient is to use a clinical end point such as rejection or graft failure (32). The high-throughput multiplex non-HLA assay described here can be used to assess defined patient groups to determine whether these 14 non-HLA antibodies are associated with graft rejection or graft loss. Recently, we determined the effect of pretransplant single antigen bead-defined donor-specific HLA antibodies in 4770 kidney transplant recipients (2). This well defined cohort will be used to determine the clinical relevance of these pretransplant non-HLA antibodies and results will be published in a separate study.

## Author Contributions

EK, TK-H, BW, AD, LP, JM, NB, and HO: Conceived and designed the experiments. EK, TK-H, AD, LP, and JM: Performed the experiments. EK, TK-H, BW, AD, LP, and JM: Analyzed the data. EK, TK-H, BW, IJ, WA, AvdM, LH, MaB, ES, CH, FvR, AvZ, MV, MiLB, AD, LP, JM, NB, MS, JS, BH, AL, LB, CR, MT, CV, LW, EvD, MG, MC, FvI, SN, NL, WS, KvdP, NvdW, ItB, FB, PvdB, JdF, MiB, SH, DR, FC, and HO: Contributed reagents, materials, and analysis tools. LH, MaB, FvR, AvZ, MV, MS, JS, EvD, MG, MC, FvI, SN, KvdP, NvdW, ItB, FB, PvdB, JdF, MiB, EK, TK-H, LH, AvZ, JM, NB, BH, LW, MC, FvI, ItB, SH, and HO: Evaluated kidney transplant patients contributed to writing of the manuscript. All authors reviewed and approved the final version of the manuscript.

### Conflict of Interest Statement

The authors declare that the research was conducted in the absence of any commercial or financial relationships that could be construed as a potential conflict of interest.

## Abbreviations

AMR: antibody-mediated rejection
APMAP: Adipocyte plasma membrane-associated protein
ARHGDIB: Rho GDP-dissociation inhibitor 2
ARHGEF6: Rho guanine nucleotide exchange factor 6
AT1R: Angiotensin type I receptor
BPIFB1: BPI fold-containing family B member 1
DSA: donor-specific anti-HLA antibodies
ETAR: Endothelin type A receptor
HLA: human leukocyte antigen
IIFT: Indirect immunofluorescence test
N-GlcNAc: N-Acetylglucosamine
PECR: Peroxisomal trans-2-enoyl-CoA reductase
PLA2R: Phospholipase A2 receptor
PRA: Panel reactive antibodies
PRKCZ: Protein kinase C zeta type
Tubb4B: Tubulin beta-4B.
