# Supplementary material for: Development and Validation of a Multiplex Non-HLA Antibody Assay for the Screening of Kidney Transplant Recipients
Source: Front Immunol. 2018 Dec 19;9:3002. doi: 10.3389/fimmu.2018.03002 (PMC6315148; doi:10.3389/fimmu.2018.03002)
Supplement: Supplementary file 1 [file Data_Sheet_1.docx]

Supplementary Material

**Development and validation of a multiplex non-HLA antibody assay for the screening of kidney transplant recipients**

Elena G. Kamburova^1^, Tineke Kardol-Hoefnagel^1^, Bram W. Wisse^1^, Irma Joosten^2^, Wil A. Allebes^2^, Arnold van der Meer^2^, Luuk B. Hilbrands^3^, Marije C. Baas^3^, Eric Spierings^1^, Cornelis E. Hack^1^, Franka E. van Reekum^4^, Arjan D. van Zuilen^4^, Marianne C. Verhaar^4^, Michiel L. Bots^5^, Adriaan C.A.D. Drop^1^, Loes Plaisier^1^, Jan Meeldijk^1^, Niels Bovenschen^1,6^, Marc A.J. Seelen^7^, Jan Stephan Sanders^7,^ Bouke G. Hepkema^8^, Annechien J. A. Lambeck^8^, Laura B. Bungener^8^, Caroline Roozendaal^8^, Marcel G.J. Tilanus^9^, Christina E. Voorter^9^, Lotte Wieten^9^, Elly M. van Duijnhoven^10^, Mariëlle A.C.J. Gelens^10^, Maarten H.L. Christiaans^10^, Frans J. van Ittersum^11^, Shaikh A. Nurmohamed^11^, Neubury M. Lardy^12^, Wendy Swelsen^12^, Karlijn A.M.I. van der Pant^13^, Neelke C. van der Weerd^13^, Ineke J.M. ten Berge^13^, Frederike J. Bemelman^13^, Paul J.M. van der Boog^14^, Johan W. de Fijter^14^, Michiel G.H. Betjes^15^, Sebastiaan Heidt^16^, Dave L. Roelen^16^, Frans H. Claas^16^, Henny G. Otten^1*^

^1^ Laboratory of Translational Immunology, University Medical Center Utrecht, Utrecht, The Netherlands

^2^ Radboud university medical center, Radboud Institute for Molecular Life Sciences, Laboratory Medicine, Laboratory of Medical Immunology, Nijmegen, The Netherlands

^3^ Radboud university medical center, Radboud Institute for Health Sciences, Department of Nephrology, Nijmegen, The Netherlands

^4^ Department of Nephrology and Hypertension, University Medical Center Utrecht, Utrecht, The Netherlands

^5^Julius Center for Health Sciences and Primary Care, University Medical Center Utrecht, Utrecht, The Netherlands

^6^ Department of Pathology, University Medical Center Utrecht, Utrecht, The Netherlands;

^7^ Department of Nephrology, University of Groningen, University Medical Center Groningen, Groningen, The Netherlands

^8^ Department of Laboratory Medicine, University of Groningen, University Medical Center Groningen, Groningen, The Netherlands

^9^ Department of Transplantation Immunology, Tissue Typing Laboratory, Maastricht University Medical Center, Maastricht, The Netherlands

^10^ Department of Internal Medicine, Division of Nephrology, Maastricht University Medical Center, Maastricht, The Netherlands

^11^ Amsterdam University Medical Center, Vrije Universiteit Amsterdam, Department of Nephrology, Amsterdam, The Netherlands

^12^ Department of Immunogenetics, Sanquin Diagnostic Services, Amsterdam, The Netherlands

^13^ Amsterdam University Medical Center, University of Amsterdam, Department of Internal Medicine, Renal Transplant Unit, Amsterdam, The Netherlands

^14^ Dutch Organ Transplant Registry (NOTR), Dutch Transplant Foundation (NTS), Leiden, The Netherlands

^15^ Department of Nephrology, Leiden University Medical Center, Leiden, The Netherlands

^16^ Department of Internal Medicine, Nephrology, Erasmus Medical Center, Rotterdam, Department of Nephrology, Rotterdam, The Netherlands

^17^ Department of Immunohematology and Blood Transfusion, Leiden University Medical Center, Leiden, The Netherlands

*** Correspondence:**Henny G. Otten
[H.G.Otten@umcutrecht.nl](mailto:H.G.Otten@umcutrecht.nl)

**Supplementary Information**

**Table of Contents**

Supplementary Table S1 4

Supplementary Table S2 5

Supplementary Table S3 6

| **Supplementary Table S1. Overview of the sequences used for the in-house protein production.** | | | |
| --- | --- | --- | --- |
|  |  |  |  |
| **Protein** | **Uniprot Feature Identifier** | **Uniprot entry and positions used** | **Remarks** |
| PLA2R | PRO_5000144349 | Q13018 [21-1397] | Only the extracellular part |
| Agrin_HaloTag | PRO_0000421614 | O00468 [1103 - 2067] | C-terminal 110 kDa subunit, isoform 6 |
| APMAP_HaloTag | PRO_0000205945 | Q9HDC9 [2 - 416] |  |
| ARHGDIB_HaloTag | PRO_0000219016 | P52566 [2 - 201] |  |
| ARHGEF6_HaloTag | PRO_0000080917 | Q15052 [2 - 776] |  |
| Endorepellin_HaloTag | PRO_0000391621 | P98160 [3687 - 4391] |  |
| ETAR_HaloTag | PRO_0000012721 | P25101 [21-80; Linker; 133-159; Linker; 230-256; Linker; 329-347] | Four extracellular domains with linkers |
| LMNB1_HaloTag | PRO_0000063816 | P20700 [2 - 586] |  |
| LPLUNC1_HaloTag | PRO_0000017180 | Q8TDL5 [2 - 484] |  |
| PLA2R_HaloTag | PRO_5000144349 | Q13018 [21-1397] | Only the extracellular part |
| PRKCZ_HaloTag | PRO_0000055701 | Q05513 [2 - 592] |  |
| Transferrin_HaloTag | PRO_5004165272 | Q06AH7 [20 - 698] |  |
| TUBB4B_HaloTag | PRO_0000048248 | P68371 [2 - 445] |  |
| Vimentin_HaloTag | PRO_0000063754 | P08670 [2 - 466] |  |

| **Supplementary Table S2. List of antibodies and their dilutions used in the Western blot.** | | | | |
| --- | --- | --- | --- | --- |
| **Antibodies** | **Company** | **Catalog #** | **Stock concentration (mg/ml)** | **Dilution used** |
| Goat anti-human **AT1R** | Abnova | PAB7122 | 0.5 | 1:1500 |
| Goat anti-**GST tag** | Novus Bio | NB600-328 | 1 | 1:5000 |
| Mouse anti-**HaloTag** | Promega | G9211 | 1 | 1:1000 |
| Mouse anti-**HIS tag** | BD | 51-9000012 | 0.5 | 1:2000 |
| Rabbit anti-**Transferrin** | Abnova | PAB16876 | 1 | 1:2000 |
| Goat anti-Mouse HRP | Jackson ImmunoResearch | 115-036-072 |  | 1:10000 |
| Rabbit anti-Goat HRP | Jackson ImmunoResearch | 305-035-003 |  | 1:10000 |
| Goat anti-Rabbit HRP | Pierce | 31460 | 0.8 | 1:10000 |

| **Supplementary Table S3. List of antibodies and their dilutions used in the Luminex assays.** | | | | | | |
| --- | --- | --- | --- | --- | --- | --- |
|  |  |  |  |  |  |  |
| **Antibodies** | **Company** | **Catalog #** | **Stock concentration (mg/ml)** | **Validated application ELISA** | **Dilution used** |  |
| Goat anti-human **Agrin** | Santa Cruz | sc-6166 | 0.2 | Yes | 1:12.5 |  |
| Goat anti-human **APMAP** (c20orf3) | Abnova | H00057136-B01P | 1 | N/A | 1:12.5 |  |
| Mouse anti-human **ARHGDIB** | Abnova | H00000397-M01 | 1 | Yes | 1:100 |  |
| Mouse anti-human **ARHGEF6** | Abnova | H00009459-B01P | 1 | N/A | 1:12.5 |  |
| Goat anti-human **AT1R** | Abnova | PAB7122 | 0.5 | Yes | 1:1.5 |  |
| Goat anti-human **Endorepellin** | R&D systems | AF2364 | 0.2 | N/A | 1:25 |  |
| Mouse anti-human **ETAR** (EDNRA) | Abnova | H00001909-M02 | 1 | Yes | 1:12.5 |  |
| Rabbit-anti human **LMNB1** | Abnova | PAB0339 | 0.5 | N/A | 1:12.5 |  |
| Rabbit anti-human **LPLUNC1** | Sino Biological | 13275-T24 | 1 | Yes | 1:12.5 |  |
| Rabbit anti-human **PECR** | GeneTex | GTX112083 | 1 | N/A | 1:250 |  |
| Goat anti-human **PLA2R** | Sigma-Aldrich | SAB2501500 | 0.5 | Yes | 1:25 |  |
| Mouse anti-human **PRKCZ** | Abnova | H00005590-M01 | 1 | Yes | 1:12.5 |  |
| Rabbit anti-human **Transferrin** | Abnova | PAB16876 | 1 | Yes | 1:12.5 |  |
| Mouse anti-human **TUBB4B** | Abnova | H00010383-A01 |  | Yes | 1:12.5 |  |
| Mouse anti-human **Vimentin** | Abnova | MAB1534 | 1 | N/A | 1:12.5 |  |
| Goat anti-rabbit PE | Jackson ImmunoResearch | 111-116-144 | 0.5 |  | 1:50 |  |
| Donkey anti-goat PE | Jackson ImmunoResearch | 705-116-147 | 0.5 |  | 1:50 |  |
| Goat anti-mouse PE | Jackson ImmunoResearch | 115-116-072 | 0.5 |  | 1:50 |  |
| Goat anti-human IgG PE | Jackson ImmunoResearch | 109-116-098 | 0.5 |  | 1:50 |  |

N/A: not available
